# Primary mammary small-cell carcinoma: A case report and review of the literature

**DOI:** 10.4103/0971-5851.56334

**Published:** 2009

**Authors:** Altaf Gauhar Haji, Shekhar Sharma, D. K. Vijaykumar, Poulome Mukherjee, R. Manoj Babu, K. Chitrathara

**Affiliations:** *Department of Surgical Oncology, Amrita Institute of Medical Sciences and Research Center, Ernakulam, Kerala, India*

**Keywords:** *Small cell carcinoma*, *mammary*, *neuroendocrine tumor*

## Abstract

Only a few cases of primary small-cell carcinoma of the breast have been documented in the current medical literature. A confident diagnosis can only be made if a nonmammary site is excluded or if an *in-situ* component can be demonstrated histologically. These criteria have been met only in a very few of the published cases, including this case report. We describe a case of a 68-year-old lady with left breast lump, which was diagnosed as breast cancer on fine-needle aspiration and core biopsy. Metastatic workup was negative for disease elsewhere, and she received 3 cycles of neoadjuvant chemotherapy followed by surgery (modified radical mastectomy). However, the disease behaved very aggressively in the postoperative period. There is a lack of consensus regarding the management of the primary tumor. Present surgical treatment options are similar to those in cases of invasive ductal breast cancer, as appropriate for the size and stage of the lesion. A review of current literature on his rare entity is also presented.

## INTRODUCTION

Small-cell carcinoma, although most commonly encountered in the lung, can occur in many extrapulmonary sites, including the salivary glands, upper respiratory mucosa, intestinal tract, pancreas, urinary tract and other organs.[1] Despite a histologically undifferentiated appearance, these tumors are notable for variable expression of neuroendocrine (NE) markers.[2]

Primary mammary small-cell carcinoma (PMSCC) is an uncommon entity and has been documented infrequently in the current medical literature.[2–14] The diagnosis of PMSCC can be made with confidence only if a nonmammary site is excluded clinically and radiologically or if an *in-situ* component can be demonstrated histologically.[4] These criteria have not been met in all of the published descriptions of this rare neoplasm. It is important to differentiate between small-cell carcinoma originating primarily in breast and metastatic disease to the breast. 

## CASE REPORT

A 68-year-old woman presented with a left breast lump that had gradually increased in size over the last 3 months. She had no other significant local or systemic symptoms or any other significant medical history. Physical examination revealed a 5.8 × 4.3 cm lump in the upper outer quadrant of left breast with multiple significantly enlarged left axillary nodes. Fine-needle aspiration and core biopsy were suggestive of infiltrating ductal carcinoma (IDC). Metastatic workup [including computed tomography (CT) chest, ultrasound abdomen and bone scans] excluded any obvious metastatic focus, and she was then started on Docetaxel and Epirubicin as a part of neoadjuvant chemotherapy protocol, with good clinical response (>50% reduction in the sum of two dimensions) after 6 cycles of chemotherapy. She then underwent modified radical mastectomy.

The gross primary tumor size at the time of surgery was 2.8 × 2.2 cm, with 13 lymph nodes dissected out during axillary dissection. In the final histopathology report, the resection margins were free of tumor and 1 (out of 13) lymph node showed metastatic tumor. Routine immunohistochemistry for Estrogen receptor (ER), Progesterone receptor (PR) and Her-2neu was reported as negative.

**Figure 1:**
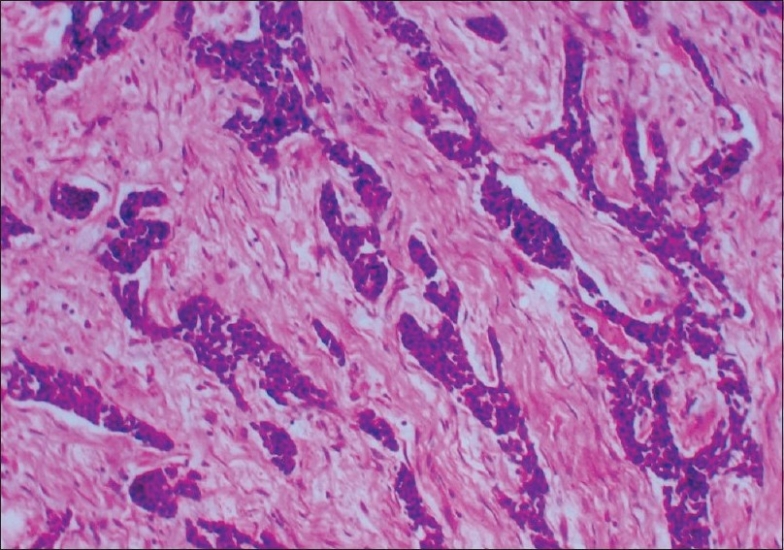
Low-power view of primary mammary cell carcinoma —H and E stain, (magnification ×100): Photomicrograph of the lesion inthe breast showing monomorphic small cells with scanty cytoplasm arranged in cribriform and trabecular pattern

**Figure 2:**
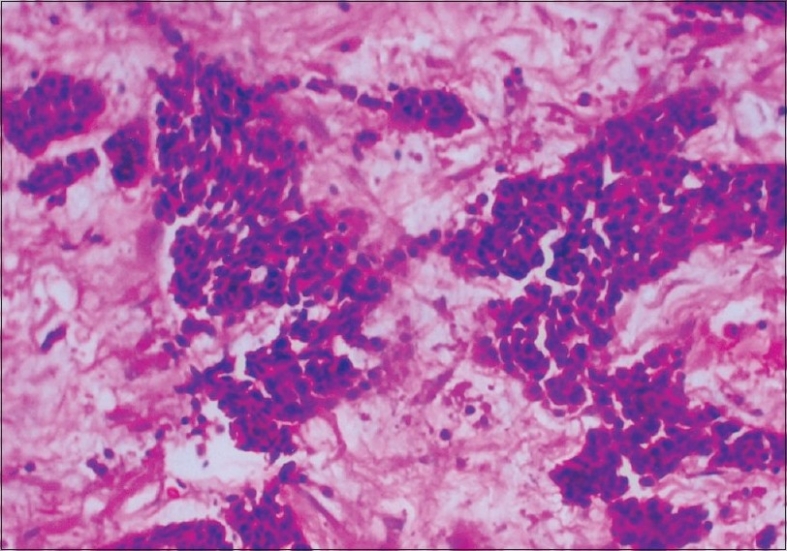
High-power view of primary mammary cell carcinoma — H and E stain, (magnification ×200): High-power magnification photomicrograph showing small cells with prominent hyperchromatic nuclei with evenly distributed chromatin, scanty cytoplasm and occasional mitosis

Microscopically, the majority of cells were small with scanty cytoplasm and hyperchromatic nuclei, admixed with occasional nucleoli arranged in nests with a focal cribriform pattern and trabeculae separated by desmoplastic stroma. Mitosis was scanty and necrosis was absent [Figures 1 and 2]. No lymphovascular or perineural invasion was seen. Two areas of *in-situ* component were also noted showing cells of similar morphology. Immunohistochemistry was negative for neuron-specific enolase, chromogranin and synaptophysin, while it was positive for cytokeratin.

A thorough evaluation to search for the extra-mammary primary site was carried out, including a CT scan of the abdomen, cervical smear and a review of the CT scan chest in the postoperative period. Surprisingly, this time the abdominal scan showed several metastatic liver deposits, mainly in segments V and VI. Results of other investigations were normal. The patient died as a result of the disease a month after surgery. 

Discussions with pathologist and review of the initial core biopsy and comparison with final histopathology were done, which revealed similar morphology of the cells. Possible reason for initial diagnosis of IDC, postulated during this discussion and review, was the first reported FNAC, clinical history and nature and site of biopsy.

## DISCUSSION

Few cases of PMSCC have been described in the available literature, which consists largely of case reports; [4–11]and 4 series: One each with nine,[3] seven,[12] three[13] and four patients.[14] The absence of a primary small-cell carcinoma elsewhere and the presence of an *in-situ* component favor the diagnosis of a primary small-cell lesion originating in the breast.[4] 

Table 1 presents the clinico-pathological findings of all published cases of primary mammary small-cell carcinoma.

**Table 1:** Clinico-pathological findings of published cases of primary mammary small-cell carcinoma

Author	Ref.no	Age	TNM	Size(cm)	LN	IS	VI	ER	Treatment	F-U	Status
Jundt	2	52	TxN1M0	NS	NS	NS	NS	NS	CT/RT	14	DOD
Shin	3	43	T1NxM0	1.3	NA	NS	NS	NS	TE/RT	30	NED
		44	T1N0M0	2	0/21	NS	*	Pos	TE/CT/RT	27	NED
		46	T2N1M0	3.4	2/26	NS	*	Pos	TE/CT	11	AWD
		50	T2NxM0	2.2	+ / ?	NS	*	Pos	TE/CT/HT	35	NED
		51	T1N0M0	1.5	0/10	NS	*	Pos	TE/RT	25	NED
		57	T2N0M0	2.5	0/19	NS	*	Pos	MRM/CT	10	NED
		62	T2N1M0	5	2/17	NS	*	Pos	NACT/MRM	32	AWD
		64	T1N0M0	1.8	0/15	NS	*	Pos	TE/CT	10	NED
		70	T2NxM0	4	+ / ?	NS	*	Pos	TE/CT/RT	3	NED
		67^a^	T1NxM0	2	+ / ?	Yes	NS	Neg	TE/?	?	?
Jochems	4	71	T2N0M0	3	0/10	No	No	Pos	MRM/HT	12	NED
Chua	5	45	T2N0M0	4.5	NS	NS	NS	Neg	TE	<1	NED
Yamasaki	6	41	T2N0M0	4.5	0/5	Yes	Yes	Neg	MRM/CT	16	NED
Francois	7	68	T2N0M0	4	0/20	No	Yes	Neg	MRM/RT	21	DOD
Fukunaga	8	56	T4NxMx	10.5	1/11	Yes	Yes	Neg	RM	48	NED
Wade	9	52	T4N1M1	10	25/25	No	Yes	Ns	MRM/CT	9	DOD
Sridhar	10	58	T1N1M0	2	1/9	NS	NS	NS	BCS/CT/RT	18	NED
Mariscal	11	53	T2N2M0	5	+ / ?	NS	NS	NS	NACT/MRM	6	NED
Yamamoto	12	53	T3N2M0	6.5	+ / ?	NS	NS	NA	Surg^@^	34	NED
		75	T2N1M0	2.5	+ / ?	NS	NS	NA	Surg^@^	43	NED
		58	T2N0M0	2.3	NS	NS	NS	NA	Surg^@^	23	NED
		65	T1N0M0	0.7	NS	NS	NS	NA	Surg^@^	28	NED
		70	T3N0M0	6.6	NS	NS	NS	NA	Surg^@^	24	NED
		50	T3N0M0	6.0	NS	NS	NS	NA	surg^@^	34	NED
		50	T1N0M0	0.5	NS	NS	NS	NA	surg^@^	35	NED
Jablon	13	72	T2N0M0	2.5	0/14	NS	NS	Pos	MRM	84	NED
		71	T1N0M0	2	0/21	NS	NS	Pos	MRM	60	NED
		54	T1N0M0	1.3	0 / ?	NS	NS	Neg	MRM	15	NED
Papotti	14	64	T1N0M0	2	0/10	Yes	Yes	Neg	RM	44	NED
		41	T2N3M0	3.5	7/14	Yes	Yes	Neg	RM/CT	15	DOD
		50	T2N1M0	3	3/18	Yes	Yes	Neg	RM/CT	14	DOD
		69	T3N1M0	5	3/10	Yes	Yes	Pos	MRM/HT	9	DOD
Present		68	T3N1M0	5.8	1/13	No	Yes	Pos	MRM	1	DOD

(This table lists out the details of 33 cases of primary mammary small-cell carcinoma published in English literature to date.) LN = Lymph node status (positive/ total number);IS = *In-situ* component; VI = Vascular invasion; ER: Estrogen receptor status; Pos = Positive; Neg: Negative; NS = Not specified; MRM = Modified radical mastectomy;RM = Radical mastectomy; TE = Tumorectomy; NACT = Neoadjuvant chemotherapy; CT = Chemotherapy; RT = Radiotherapy; F-U = Follow-up; DOD = Dead of disease;NED = No evidence of disease; AWD = Alive with disease;

* = Present in 7 cases; ? = Not known or not reported; + = Involved; NA = Not available; Surg^@^ = Surgery done but exact details not known; BCS = Breast-conserving surgery

a = Associated with Paget's disease, reported as an addendum to the initial series of 9 cases

### Clinical presentation 

There are no notable differences in presentation when compared with other types of breast cancers. The average (mean) age at presentation of the reported patients [Table 1] is 57.6 years (range, 41-75 years). Patients often present with a palpable mass (mean size of 3.5 cm; range, 0.5-10.5 cm), which usually appears circumscribed on mammography and ultrasound examination. 

### Histology 

These tumors exhibit features similar to those of NE tumors of the gastrointestinal tract or lung. Although they exhibit NE markers in more than 50% of the cell population, there is no consistent immunoreactivity, and the diagnosis should not be excluded only on the basis of negative reactivity for selected NE markers.[2] Thus, at present, the demonstration of an NE immunoprofile is supportive but not essential for the diagnosis of PMSCC.[3,4]

In our patient, the tumor had histological characteristics of a small-cell carcinoma, including 'small' (approximately the sum of diameters of three lymphocytes) nuclei with scant cytoplasm; finely granular, evenly distributed chromatin; absent or inconspicuous nucleoli; and frequent mitoses. The observations of variable immunoreactivity provide an explanation for the negative immunoreactivity for synaptophysin, neuron-specific enolase and chromogranin in our case.

### Molecular biology

It is generally agreed that tumors of the breast with endocrine features do not arise from preexisting endocrine cells.[15] The immunoprofile suggests that small-cell carcinoma of the breast may represent a type of 'metaplasia.' Hoang *et al*.,[16] reported identical molecular alterations at multiple chromosomal regions in 2 cases of PMSCC using laser-capture micro-dissection followed by loss-of-heterozygosity (LOH) analysis. Further research is necessary to analyze the molecular pathology of PMSCC.

### Imaging 

The existence of a primary lesion is controversial and should only be considered after a thorough evaluation has been performed to rule out an occult primary tumor elsewhere. A thorough history-taking and detailed clinical evaluation followed by radiological investigations (CT chest and CT abdomen at the minimum) are recommended before a diagnosis of primary mammary small-cell carcinoma can be made.[3] This is in contrast to invasive epithelial breast cancer, where a metastatic workup is recommended only for locally advanced cases; and that too includes only a chest X-ray, USG abdomen and bone scan.

### Treatment

The recognition of a presumptive origin of PMSCC is extremely important because of the different clinical management of a primary versus a metastatic tumor[16,17] (see present consensus below).

Surgical options, used in the reported 34 cases, vary widely from no surgery (1 case); plain tumor excision (9 cases); breast-conserving surgery (1 case); modified radical mastectomy (12 cases) to even radical mastectomy (4 cases). Details of surgery are in fact not available for 7 cases.

There are no recommendations or guidelines for chemotherapy or radiotherapy for PMSCC. This lack of consensus is quite apparent on review of treatment schedules used in the reported cases. 

In the largest series reported, Shin *et al*.,[3] treated 7 patients with chemotherapy, although the authors do not provide any further details of the chemotherapy that these patients received. Yamamoto *et al*.,[12] treated 3 patients with adjuvant chemotherapy (cyclophosphamide, adriamycin and fluorouracil ‒ CAF). Papotti *et al*.,[14] treated 1 out of their 4 patients with adjuvant Streptozocin initially; and later, with CAF for recurrence. Sridhar *et al*.,[10] used cisplatin and adriamycin-based chemotherapy; Francois *et al*.,[7] used a combination of cyclophosphamide, adriamycin and etoposide; Wade *et al*.,[9] used etoposide initially, followed by cyclophosphamide, adriamycin and vincristine for recurrence; while Mariscal *et al*.,[11] used cisplatin and etoposide. Jundt *et al*.,[2] used adjuvant chemotherapy but have not reported on the details.

Similarly, adjuvant radiotherapy is reported only in 8 out of 34 cases, with minimal or no details regarding the fields, portals, dosage, fractions, etc. Thus, there is paucity of clear recommendations regarding management of these rare tumors. The present consensus is that a patient with PMSCC should be treated in a manner similar to that used to treat invasive epithelial breast carcinoma, as appropriate for the size and stage of the lesion; although there is no definitive evidence that this approach is superior to, or more effective than, other approaches. 

### Prognosis

Historically these tumors are considered highly malignant with a very poor prognosis. It was generally thought that the prognosis of these tumors was as poor as their counterpart in the lung.[4,9] This, however, may not be a true representation of the natural history of the disease. Available literature seems to suggest that the prognosis may not be as grave as believed, especially for tumors detected earlier and those without lymph node metastasis. With a mean follow-up period of nearly 2 years (mean follow-up period, 23.9 months; range, 1-84 months), literature shows a mortality of 20.5% (7 out of 34 cases). 

## CONCLUSION

PMSCC is extremely rare, and historically it is considered to be a highly aggressive tumor. The initial diagnostic tools (mammography, fine-needle aspiration cytology and core biopsy) may not detect this malignant tumor in many instances. Most of the cases are treated according to conventional clinical and pathological criteria, similar to those for the treatment of other invasive breast cancers. It is important to rule out extra-mammary primary sites, as PMSCC is treated as an invasive epithelial breast cancer. The prognosis remains unclear, although more recent data suggest that the prognosis for relatively early-stage small-cell carcinoma may be more favorable than historically believed.
